# The Yarrow Convalescent Home

**Published:** 1898-02-26

**Authors:** 


### THE YARROW CONVALESCENT HOME.

MR. F. MYERS, Secretary of the Yarrow Home, writes: You were kind enough in your issue of November 2nd, 1895, to publish a description of the Yarrow Home for Convalescent Children at Broadstairs. I have the pleasure to report that since the opening of this institution a little over two years ago no less than 1,299 children have been benefited by a stay there. It will be remembered the home is fully endowed and no subscriptions are required, also that it is not intended for the very poor, but for children who have been respectably brought up and whose parents cannot afford to send them to the seaside entirely at their own expense. During the summer months the applications for admission are far in excess of the capabilities of the home, but in the winter it is only partly filled, and the trustees are most anxious to maintain its utility equally throughout the year. There is little doubt that the falling off in applications during the winter may arise from the fact that the home is thought not to be a very desirable resort during the inclement part of the year, but such is not the case. The building is thoroughly warmed and ventilated, a healthy temperature being maintained throughout; and there are play corridors within and covered playgrounds without. The grounds are planted and well sheltered, and there are but very few days in the year when children cannot enjoy the fresh air even when the weather debars them from going to the sea-front.

